# Supplementary material for: VOCs from Exhaled Breath for the Diagnosis of Hepatocellular Carcinoma
Source: Diagnostics (Basel). 2023 Jan 10;13(2):257. doi: 10.3390/diagnostics13020257 (PMC9858163; doi:10.3390/diagnostics13020257)
Supplement: Supplementary file 1 [file diagnostics-13-00257-s001.zip › diagnostics-2105835-supplementary.pdf]

diagnostics

MDPI

Supplemental Material

VOCs from Exhaled Breath for the Diagnosis of Hepatocellular Carcinoma

Supplemental Table S1. VOC level at pre- and post-treatment of 38 HCC patients.

VOCs	Pre-treatment*	Post-treatment*	Mean difference between post-& pre-treatment (95% CI)	*p*
Ethanol	0.25 (0.17, 0.36)	0.24 (0.15, 0.47)	-0.01 (-0.04, 0.02)	0.709
Acetone monomer	3.84 (2.44, 4.73)	3.91 (2.82, 4.82)	0.16 (-0.08, 0.41)	0.221
Dimethyl sulfide	0.67 (0.17, 1.39)	0.48 (0.15, 1.38)	-0.17 (-0.29, -0.05)	0.007
1,4-pentadiene	0.31 (0.11, 1.49)	0.24 (0.10, 0.92)	-0.08 (-0.19, 0.03)	0.156
Benzene	0.15 (0.06, 0.72)	0.11 (0.03, 0.45)	-0.07 (-0.13, -0.02)	0.013
Isopropyl alcohol	0.16 (0.10, 1.06)	0.13 (0.08, 0.76)	-0.05 (-0.12, 0.02)	0.162
Acetone dimer	5.10 (2.98, 5.83)	4.35 (2.70, 5.33)	-1.06 (-1.40, -0.71)	<0.001
Acetonitrile	0.15 (0.10, 3.67)	0.10 (0.06, 0.40)	-0.05 (-0.08, -0.01)	0.006
Toluene	0.41 (0.14, 2.33)	0.38 (0.21, 7.14)	-0.101 (-0.24, 0.03)	0.130

*Data are shown as median (range) in arbitrary unit (AU).

Supplemental Table S2. Change in VOC level in responder and non-responder after HCC treatment*.

VOCs	Response group	Non-response group	*p*
Ethanol	-0.03 (-0.16, 0.15)	0.24 (-0.57, 1.47)	0.137
Acetone monomer	-0.17 (-0.95, 2.05)	0.02 (-0.09, 0.25)	0.497
Dimethyl sulfide	-0.15 (-0.71, 0.32)	0.05 (-0.28, 0.32)	0.031
1,4-pentadiene	-0.24 (-0.85, 0.19)	0.05 (-1.05, 0.48)	0.052
Benzene	-0.04 (-0.62, 0.36)	-0.08 (-0.34, 0.15)	0.286
Isopropyl alcohol	0.01 (-0.08, 0.02)	-0.02 (-0.30, 0.00)	0.383
Acetone dimer	-1.32 (-2.75, -0.06)	-0.12 (-2.63, 1.80)	0.003
Acetonitrile	-0.06 (-0.18, 0.14)	-0.03 (-0.16, 0.07)	0.083
Toluene	-0.07 (-2.08, 0.19)	0.02 (-0.30, 0.52)	0.121

*Data are shown as median (range) in arbitrary unit (AU).
